# Effect of dietary krill oil supplementation on the endocannabinoidome of metabolically relevant tissues from high-fat-fed mice

**DOI:** 10.1186/1743-7075-8-51

**Published:** 2011-07-13

**Authors:** Fabiana Piscitelli, Gianfranca Carta, Tiziana Bisogno, Elisabetta Murru, Lina Cordeddu, Kjetil Berge, Sally Tandy, Jeffrey S Cohn, Mikko Griinari, Sebastiano Banni, Vincenzo Di Marzo

**Affiliations:** 1 Endocannabinoid Research Group, Istituto di Chimica Biomolecolare, CNR, Pozzuoli (NA), Italy; 2 Dipartimento di Biologia Sperimentale, Università di Cagliari, Italy; and Nutrisearch s.r.l. Pula (CA) Italy; 3 Aker BioMarine ASA, Oslo, Norway; 4 Nutrition and Metabolism Group, Heart Research Institute, Sydney, Australia; 5 Clanet Oy, Helsinki, Finland

## Abstract

**Background:**

Omega-3 polyunsaturated fatty acids (ω-3-PUFA) are known to ameliorate several metabolic risk factors for cardiovascular disease, and an association between elevated peripheral levels of endogenous ligands of cannabinoid receptors (endocannabinoids) and the metabolic syndrome has been reported. We investigated the dose-dependent effects of dietary ω-3-PUFA supplementation, given as krill oil (KO), on metabolic parameters in high fat diet (HFD)-fed mice and, in parallel, on the levels, in inguinal and epididymal adipose tissue (AT), liver, gastrocnemius muscle, kidneys and heart, of: 1) the endocannabinoids, anandamide and 2-arachidonoylglycerol (2-AG), 2) two anandamide congeners which activate PPARα but not cannabinoid receptors, *N*-oleoylethanolamine and *N*-palmitoylethanolamine, and 3) the direct biosynthetic precursors of these compounds.

**Methods:**

Lipids were identified and quantified using liquid chromatography coupled to atmospheric pressure chemical ionization single quadrupole mass spectrometry (LC-APCI-MS) or high resolution ion trap-time of flight mass spectrometry (LC-IT-ToF-MS).

**Results:**

Eight-week HFD increased endocannabinoid levels in all tissues except the liver and epididymal AT, and KO reduced anandamide and/or 2-AG levels in all tissues but not in the liver, usually in a dose-dependent manner. Levels of endocannabinoid precursors were also generally down-regulated, indicating that KO affects levels of endocannabinoids in part by reducing the availability of their biosynthetic precursors. Usually smaller effects were found of KO on OEA and PEA levels.

**Conclusions:**

Our data suggest that KO may promote therapeutic benefit by reducing endocannabinoid precursor availability and hence endocannabinoid biosynthesis.

## Introduction

The endocannabinoid system, and in particular the cannabinoid receptor type 1 (CB_1_), are profoundly involved in the regulation of energy balance, at both the central and peripheral levels [1,2]. Apart from CB_1_, this system includes the cannabinoid receptor type 2 (CB_2_) [3,4], the endogenous agonists, *N*-arachidonoylethanolamine (anandamide, AEA) and 2-arachidonoyl glycerol (2-AG), known as endocannabinoids (ECs) [5-7], and enzymes for EC biosynthesis and degradation [8-11]. ECs are nominally synthesized from arachidonic acid (AA), but are phospholipid (PL)-derived mediators. AEA and 2-AG are synthesized, respectively, through the *N*-acylphosphatidylethanolamine-selective "phospholipase D" (NAPE-PLD), among others, and the diacylglycerol lipases (DAGL). The main enzymes for EC degradation are fatty acid amide hydrolase (FAAH) and monoacylglycerol lipase (MAGL), for AEA and 2-AG, respectively. NAPE-PLD can also produce other *N*-acylethanolamines (NAE), like *N*-palmitoylethanolamine (PEA) and *N*-oleoylethanolamine (OEA), from the corresponding *N*-acylphosphatidylethanolamines, and these two compounds are also good substrates for FAAH [12]. PEA and OEA do not bind CB_1 _and CB_2 _receptors, but act via other receptor types, such as the peroxisome proliferator-activated receptor-α (PPAR-α) [13] and the transient receptor potential vanilloid type-1 (TRPV1) channel [14].

The beneficial effects of omega-3 polyunsaturated fatty acids (ω-3 PUFAs) on the risk of developing cardiovascular diseases (CVD) [15] and metabolic disorders, such as insulin resistance, fatty liver and hypertension [16], have been extensively studied. The most studied ω-3 PUFAs are eicosapentaenoic acid (EPA, 20:5) and docosahexaenoic acid (DHA, 22:6), present in the diet as such or biosynthesized from α-linolenic acid (ALA, 18:3) [17]. In contrast, derivatives of ω-6 PUFAs, originated from linoleic acid (LA, 18:2), and of which the major exponent is AA (ω-6 20:4), are known for their pro-inflammatory and other disease-propagating effects [18]. Both ω-3 and ω-6 PUFAs precursors are essential fatty acids because they cannot be synthesized *de novo *in mammals and have to be obtained through the diet or from dietary precursors. Major sources of ω-3 PUFAs are fish oils, whereas other types of oil, such as flaxseed oil, are a good source of ALA [19]. Krill oil (KO) is a relatively new source of ω-3 PUFAs, extracted from Antarctic krill (*Euphasia superba*) [20], a marine crustacean with the most abundant biomass on earth [21]. The chemical composition of KO is unique. It is rich in ω-3 PUFAs in the form of PL rather than triglycerides (TG) and this may influence the bioavailability and tissue incorporation of ω-3 PUFAs [22]. Moreover, KO contains a powerful antioxidant, astaxanthin, a lipid-soluble pigment that preserves KO from oxidation. This special composition possibly underlies in part the health-promoting effects of KO, such as its anti-inflammatory and hypolipidemic properties in humans [23,24].

The levels of the ECs and their lipid congeners, in peripheral tissues controlling energy homeostasis, are altered in obese animals and humans, particularly when increased adiposity is the consequence of a prolonged high fat (HF) diet, and this alteration contributes to obesity-related metabolic disorders [25]. The dysregulation of EC concentrations can be due in part to changes in the activity and/or expression of EC biosynthetic enzymes, but also to alterations in EC ultimate PL biosynthetic precursors, which in turn are sensitive to quantitative and qualitative changes in dietary fats. It was reported that dietary ω-3 PUFAs in rats can alter NAE levels in several tissues [26], whereas, in vitro, AA, DHA and EPA influence the AA content of PL in isolated mouse adipocytes, thereby modulating the levels of EC biosynthetic precursors and of ECs in these cells [27]. On the other hand, increased dietary fat in mice is well known to lead not only to obesity and increased adiposity, but also to several metabolic sequelae, such as insulin resistance, dyslipidemia and increased expression in hepatocytes of sterol regulatory element binding protein 1c (SREBP-1c), and of its target proteins acetyl-CoA-carboxylase-1 (ACC1) and fatty acid synthase (FAS), with subsequent enhanced hepatic de novo lipid biosynthesis [28]. These effects are due in part to elevation of peripheral EC levels and subsequent over-activation of CB_1_, since CB_1 _knock-out mice, or mice chronically treated with CB_1 _antagonists, exhibit little or no response to high fat diets in terms of body weight gain and increased adiposity, fatty liver, insulin resistance and dyslipidemia [28]. Importantly, dietary KO was recently shown to reduce fatty liver and systemic inflammation in obese Zucker rats through the negative modulation of EC and EC PL precursor levels [29].

We have recently observed that a shorter duration (8 instead of 14 weeks) of a certain type of HF diet with reduced levels of ω-3 and, particularly, ω-6 PUFA fatty acid precursors, and higher cholesterol levels, instead *decreases *hepatic FAS and ACC1 expression in mice, with no change in SREBP1 expression nor in plasma glucose, non-esterified fatty acids (NEFA) and adiponectin levels [30]. This regimen, however, still causes fatty liver, increased adiposity and hypercholesterolemia, all of which are still attenuated by increasing levels of dietary KO. In the present study, we aimed at verifying whether this HF diet with a different fat composition and a shorter duration with respect to that previously used [28], may have affected EC tissue levels in a different way, and, subsequently, if the beneficial effects caused by KO in this case [30] were still due to normalization of EC dysregulation. Moreover, by using sensitive lipidomic approaches based on either single-quadrupole MS or high resolution mass spectrometric (MS) and MS-MS detection [31], both combined with liquid chromatography, we analyzed, under the different dietary regimens, the levels of EC biosynthetic precursors, as well as of anandamide congeners, OEA and PEA.

## Methods

### Animals

Male C57BL/6 mice at 6 weeks of age were obtained from the Australian Resources Centre (Perth, Australia). Experiments were approved by the Animal Welfare Committee of the Sydney SouthWest Area Health Service. They were housed in standard cages (five mice per cage) at a constant temperature of 20°C with a 12 h light/dark cycle. They were allowed ad libitum access to diet and water. After 1 week of acclimatization, they were divided into five groups (two cages for each group). After treatments and sacrifice, the ATs (inguinal and epididymal), liver, gastrocnemius muscle, kidneys and heart were removed and immersed into liquid nitrogen, to be stored at -70° until extraction and purification of AEA, 2-AG, PEA, OEA and their direct biosynthetic precursors.

### Treatments

Animals were divided into 5 groups:

✔ first group of n = 6-10 mice was fed a normal nonpurified diet (N), composed of normal mice pellet (Speciality Feeds, Glen Forrest, Western Australia), containing 4.6% total fat, 4.8% crude fiber and 19% protein (see *Diets*);

✔ second group of n = 6-10 mice was fed a HF diet, containing 21 wt% butterfat and 0.15 wt% cholesterol (SF00-219, Speciality Feeds, WA) (see *Diets*);

✔ third, fourth and fifth groups of n = 6-10 mice, respectively, were fed a high-fat diet with increasing doses of KO (HFKO 1.25, 2.5 and 5), receiving 1.25, 2.5 or 5% wt of KO, respectively. KO (Superba krill oil) was prepared and analyzed by Nutrizeal (Nelson, New Zealand) and supplied by Aker Biomarine (Oslo, Norway) (see *Diets*).

### Diets

Macronutrient composition and fatty acid profiles of the diets are depicted in Tables 1 and 2. The KO used in this study contained 12.5% wt EPA, 7% wt DHA and 25% wt total omega-3 fatty acids. Lipid classes in KO were: 23% wt triglycerides, 6% wt free fatty acids, 58% wt phospholipids and 6% wt lysophospholipids [30].

**Table 1:** Nutrient composition of the normal and high fat diets used in this study, and of the high fat diet (HFD) supplemented with increasing doses (1.25, 2.5 and 5%) of krill oil (KO)

	control diet			HFD			HFD+1.25 KO			HFD+2.5 KO			HFD+5 KO		
	**macro/100g**	**en/100g**	**en%**	**macro/100g**	**en/100g**	**en%**	**macro/100g**	**en/100g**	**en%**	**macro/100g**	**en/100g**	**en%**	**macro/100g**	**en/100g**	**en%**

Protein	20.0	80.0	22.2	19.0	76.0	16.4	19.0	76.0	16.0	19.0	76.0	15.6	19.0	76.0	14.9

Fat	4.8	43.2	12.0	21.0	189.0	40.8	22.3	200.3	42.2	23.5	211.5	43.5	26.0	234.0	46.0

Carbohydrate	59.4	237.6	65.9	49.6	198.4	42.8	49.6	198.4	41.8	49.6	198.4	40.8	49.6	198.4	39.0

total	84.2	360.8	100.0	89.6	463.4	100.0	100.3	474.7	100.0	101.5	485.9	100.0	104.0	508.4	100.0

Abbreviations: Macro: macronutrient; en, energy.

**Table 2:** Fatty acid compositions of the normal and high fat diets used in this study, and of the high fat diet (HFD) supplemented with increasing doses (1.25, 2.5 and 5%) of krill oil (KO)

Composition	control diet	HFD	HFD+1.25 KO	HFD+2.5 KO	HFD+5 KO
Fatty Acid	g/100g diet	g/100g diet	g/100g diet	g/100g diet	g/100g diet
12:0		1.80	1.80	1.81	1.82

14:0	0.03	2.60	2.67	2.74	2.87

16:0	0.50	7.00	7.19	7.39	7.78

18:0	0.14	2.40	2.43	2.47	2.54

20:0	0.00	0.00	2.41	2.41	2.42

16:1	0.01	0.40			

18:1	1.90	5.50	5.90	6.31	7.11

20:1	0.03	0.00			

18:2n6	1.30	0.40	0.80	1.19	1.99

18:3n3	0.30	0.20	0.25	0.30	0.41

18:4n3	0.00	0.00	0.01	0.03	0.06

20:4n6	0.01	0.09	0.09	0.09	0.09

20:5n3	0.02	0.05	0.10	0.16	0.26

22:6n3	0.05	0.03	0.05	0.08	0.13

Cholesterol	0.00	0.15	0.15	0.15	0.15

grams	4.29	20.45	23.55	24.80	27.30

tot sat	0.67	13.80	16.51	16.81	17.42

tot MUFA	1.94	5.90	5.90	6.31	7.11

tot PUFA	1.68	0.60	1.13	1.66	2.71

n-6	1.31	0.40	0.80	1.20	1.99

n-3	0.37	0.20	0.33	0.46	0.72

ratio LA/ALA	4.30	2.00	3.20	3.90	4.90

Ratio ARA/EPA+DHA	0.10	1.10	0.60	0.40	0.20

Abbreviations: ALA, α-linolenic acid (18:3n3); ARA, arachidonic acid (20:4n6); DHA, docosahexanoic acid (C22:6n3); EPA, eicosapentaenoic acid (C20:5n3); LA, linoleic acid (18:2n6).

### Synthesis of 1-palmitoyl-2-oleoyl-N-heptadecanoyl phosphatidylethanolamine

We prepared 1-palmitoyl-2-oleoyl-N-heptadecanoyl phosphatidylethanolamine (NHPE), to be used as internal standard, by the reaction of heptadecanoyl chloride with 2-fold molar excess of 1-palmitoyl-2-oleoyl-sn-glycero-3-phosphoethanolamine (POPE, Alexis Biochemicals). Reaction was conducted in dichloromethane at 25°C for 2 h using triethylamine (Sigma-Aldrich) as a catalyst [32]. The product was fractionated by silica-gel thin-layer chromatography (chloroform-methanol-ammonia, 85:15:1, v/v/v) and characterized by ^1^H nuclear magnetic resonance spectroscopy. Purity was >99%.

### Extraction and pre-purification procedures and measurement of AEA, 2-AG, OEA and PEA levels

Extraction, purification and quantification of AEA, 2-AG, PEA and OEA from tissues require several biochemical steps as described previously [27]. N = 5 mice were used for these measurements. First, tissues were dounce-homogenized and extracted with chloroform/methanol/Tris-HCl 50 mM pH 7.5 (2:1:1, v/v) containing internal deuterated standards for AEA, 2-AG, PEA and OEA quantification by isotope dilution ([^2^H]_8_AEA, [^2^H]_5_2AG, [^2^H]_4 _PEA, [^2^H]_4 _OEA (Cayman Chemicals, MI, USA), as well as 1,2-heptadecanoin (Larodan AB, Malmo, Sweden), and 1-palmitoyl-2-oleoyl-*N*-heptadecanoyl phosphatidylethanolamine) for DAG and *N*-acyl-phosphatidylethanolamine measurement, respectively (see below). The lipid-containing organic phase was dried down, weighed and pre-purified by open bed chromatography on silica gel. Fractions were obtained by eluting the column with 99:1, 90:10 and 50:50 (v/v) chloroform/methanol. The 90:10 fraction was used for AEA, 2-AG, PEA and OEA quantification by liquid chromatography-atmospheric pressure chemical ionization-mass spectrometry (LC-APCI-MS), as previously described and using selected ion monitoring at M + 1 values for the four compounds and their deuterated homologues, as described in [1,31].

### Enzymatic digestion of 50/50 fractions

Purified 50:50 (v/v) fractions eluted from silica gel column, and containing *N*-acyl-phosphatidylethanolamines, were digested with 100 units of *S. chromofuscus *phospholipase D (Sigma-Aldrich) in ethylic ether and Tris-HCl 50 mM for 2 h at 37°C. After incubation, samples were purified by open-bed chromatography on silica gel and the column was eluted first with chloroform and then with 90:10 (v/v) chloroform/methanol. The 90:10 fractions, enriched in NAEs released from the corresponding *N*-acyl-phosphatidylethanolamines, were analysed by LC-APCI-MS as described above [1,31], by using a Shimadzu HPLC apparatus (LC-10ADVP) coupled to a Shimadzu (LCMS-2020) quadrupole MS via a Shimadzu APCI interface. *N*-acyl-phosphatidylethanolamine-derived NAEs were quantified against *N*-heptadecanoylethanolamine released from 1-palmitoyl-2-oleyl-*N*-heptadecanoyl phosphatidylethanolamine, the internal standard synthesized as described above, for which a calibration curve had been constructed in pilot experiments, and re-run occasionally, with no observed significant changes.

### LC-MS-IT-TOF analysis

**Figure 1:**
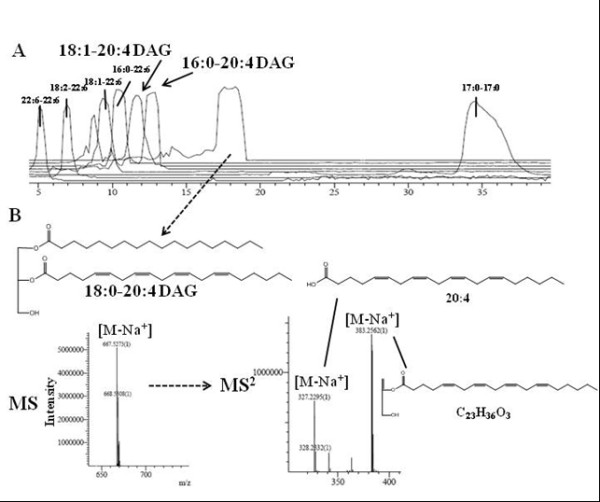
**Representative example of targeted profiling of diacylglycerols (DAGs) in lipid extracts using multi-dimensional mass spectrometry with LC-ESI-IT-ToF**. **A**: Representative extracted ion chromatogram of a pre-purified liver lipid extract containing various DAG species (22:6-22:6, 18:2-22:6, 18:1-22:6, 16:0-22:6), including the biosynthetic 2-AG precursors that were measured here (18:1-20:4, 16:0-20:4, 18:0-20:4). **B**: Example of a positive-ion electrospray mass spectrum of a major component of the extract, the 18:0-20:4 DAG precursor ion (*m/z *667.5273), as a sodium adduct, and the corresponding product ions for the CID of the fragment with *m/z *667.5273 in the MS^2 ^spectra (*m/z *327.2295 and 383.2562), which in turn correspond to the sodiated 20:4 acid and C_23_H_36_O_3 _fragments, respectively.

We measured DAG levels by LC-MS-MS using an LC20AB coupled to a hybrid detector IT-TOF (Shimadzu Corporation, Kyoto, Japan) equipped with an ESI interface. N = 3-5 mice were used for these measurements. DAGs measured included 18:1-20:4, 18:0-20:4 and 16:0-20:4 (Figure 1) and the amounts of these species in tissues, quantified using an external standard, 1,2-heptadecanoin (17:0-17:0, for which a calibration curve had been constructed in pilot experiments for the most abundant DAG species, 18:0-20:4, and considered valid also for the other DAG species), were expressed as pmols per mg of wet tissue weight. The calibration curve was also re-run occasionally, with no observed significant changes. All molecular and fragment ions were sodiated and hence provide high-resolution values for M + Na^+^. As most 20:4-containing DAG species are present in tissues in trace amounts, in some cases we had to overload the column to be sure to detect these molecules. We acquired full-scan MS^n ^spectra of selected DAG precursor ions by multiple reaction monitoring (MRM), extracted the chromatograms of the high-resolution M + Na^+ ^values and used the latter chromatograms for calibration and quantification. The limit of detection (LOD) of 17:0-17:0 was 10 pmol in MS analysis.

#### HPLC parameters

DAGs were separated using a DiscoveryC18 column (15 cm × 2.1 mm, I.D. 5 μm; Supelco) and eluted with an isocratic flow of acetonitrile:2-propanol (85:15). LC20AB HPLC pumps were used to deliver solvent at a flow rate of 200 μl/min. The samples were loaded into a 10 μl injection loop using a rheodyne valve (Shimadzu Corporation, Kyoto, Japan).

#### Mass parameters

Electrosprayed ions were generated using a capillary voltage of 4.66 kV. A curved desolvation line (CDL) was set at a temperature of 250°C to aid desolvation and a heat block temperature of 220°C was also used. To help nebulisation of the electrospray solution nitrogen was pumped into the ion source at a rate of 1.5 L/min. Ions are extracted from the ion source and focused by the Q-array device, before axial injection into the ion trap. When ions are introduced no RF potential was applied to the ion trap section. Once trapped the ions are cooled using argon from a pulse valve. The ToF mass analyser was used to acquire data in both MS and MS/MS modes. In the MS mode, a 10 msec ion accumulation time was used before ion trapping. Ions are cooled for 20 msec before ion ejection into the ToF mass analyzer. Before MS-MS analysis precursor ions can be isolated using filtered noise field (FNF) method to generate a broadband signal of frequency and it is applied to the electrodes of the ion trap causing the resonant ejection of all unwanted ions. In the MS-MS mode, instead, the ion accumulation time was 100 msec and the window used for precursor ion isolation corresponds to a width of 3 amu and 20 msec. To induce fragmentation of the precursor ion a supplementary AC (alternative current) potential was applied to the end-cap electrodes to induce resonant excitation. Argon is used as a collision gas during collision-induced dissociation (CID) and it was carried out over 30 msec using a *q *value of 0.251 (45 kHz). Three scans were accumulated in each MS-MS spectrum. In both MS and MS-MS mode data were acquired over a mass range of 200-700 m/z. In both regimes of operation ions are pulsed into the ToF with an accelerating potential of 9 kV and the detector voltage is set at 1.7 kV.

### Statistical analysis

Data are expressed as means ± SEM. To determine statistical significance, ANOVA followed by the Bonferroni's test was used for comparisons of EC and their biosynthetic precursors levels between groups. P values lower than 0.05 were considered significant.

## Results

### Effect of high fat (HF) diet on metabolic parameters and effect of krill oil (KO) on HF-induced alterations

As reported previously [30] after 8-week of HF diet, mice gained significantly more body weight than N mice, but there was no significant difference between HF mice and HFKO groups (Table 3). However, the HF diet resulted in an increase of liver weight, which was significantly reduced by KO supplementation. KO had beneficial effect also on HF diet-induced hepatomegaly by reducing total hepatic fat content in a dose-dependent manner. HF increased liver TG and cholesterol, and these values were also reduced by KO supplementation. Furthermore, the HF diet increased serum TG, cholesterol and PL levels. Dietary KO significantly decreased serum cholesterol and PL, but not TG levels. Although no difference were observed between N *vs *HF groups in serum glucose, insulin and adiponectin levels, KO supplementation decreased serum glucose, although only at the highest dose, and increased serum adiponectin levels (HF *vs *HFKO5). Moreover, as reported previously, levels of hepatic TNFα were measured and found to be significantly elevated in HF mice *vs *N mice [30]. KO supplementation reduced TNFα expression in a significantly manner with the highest dose given.

**Table 3:** Metabolic parameters and expression of some hepatic metabolic genes (*italics*) in mice fed the experimental diets

	N	HF	HFKO1.25	HFKO2.5	HFKO5
Initial weight (g)	18.1 ± 0.3	18.5 ± 0.8	18.0 ± 0.3	18.2 ± 0.6	18.3 ± 0.8

Final weight (g)	24.6 ± 0.3	31.7 ± 0.9^###^	31.0 ± 0.9	31.8 ± 0.7	32.4 ± 1.0

Weight gain	6.5 ± 0.4	13.3 ± 1.1^###^	13.0 ± 0.9	13.6 ± 0.7	14.1 ± 0.6

TG (mmol/L)	0.4 ± 0.03	0.6 ± 0.06^##^	0.8 ± 0.08	0.8 ± 0.09	0.8 ± 0.06

Cholesterol (mmol/L)	2.6 ± 0.1	4.3 ± 0.3^###^	3.4 ± 0.1^a^	3.0 ± 0.1^b^	3.0 ± 0.2^b^

PL (mmol/L)	1.3 ± 0.1	1.7 ± 0.1^###^	1.5 ± 0.1^a^	1.3 ± 0.1^c^	1.3 ± 0.1^c^

Glucose (mmol/L)	11.5 ± 0.6	13.1 ± 1.6	8.3 ± 0.6^b^	8.7 ± 0.8^a^	7.5 ± 0.8^b^

Insulin (mmol/L)	1.1 ± 0.3	0.7 ± 0.1	0.6 ± 0.1	0.8 ± 0.1	0.7 ± 0.1

Adiponectin (μg/ml)	4.7 ± 0.2	5.0 ± 0.1	6.5 ± 0.3	6.5 ± 0.4	7.5 ± 0.6^a^

*Fatty acid synthase*	*109 ± 15*	*44 ± 9*^*##*^	*27 ± 4*	*13 ± 1*^*c*^	*17 ± 2*^*c*^

*Acetyl-CoA carboxylase*	*104 ± 10*	*67 ± 10*^*##*^	*50 ± 5*	*28 ± 3*^*c*^	*32 ± 4*^*b*^

*Stearoyl-CoA desaturase*	*106 ± 11*	*232 ± 43*^*##*^	*154 ± 18*	*55 ± 8*^*c*^	*78 ± 16*^*c*^

*CD36*	*101 ± 5*	*232 ± 28*^*###*^	*256 ± 11*	*131 ± 13*	*219 ± 38*

*Monoacylglycerol lipase*	*102 ± 7*	*146 ± 17*^*#*^	*114 ± 9*	*50 ± 4*^*c*^	*68 ± 7*^*b*^

Data are taken from (30) and are means ± SEM of separate determinations in N = 6-10 mice. ^## ^P < 0.01 and ^### ^P < 0.001 N vs HF group; ^a ^P < 0.05, ^b ^P < 0.01 and ^c ^P < 0.001 HF vs HFKO groups. Expression values represent mRNA levels expressed as a ratio relative to cyclophilin, and then expressed as percent of those in N mice. Results represent means ± SEMs (n = 6-10 mice per group). Mice were fed diets for 8 weeks. Significant difference between N and HF by Student's t-test: ^#^P < 0.05, ^##^P < 0.01, and ^###^P < 0.001. Significant difference between HF- and HFKO-supplemented animals by ANOVA followed by Tukey's test for significance. ^a^, P < 0.05; ^b^, P < 0.01, and ^c^, P < 0.001. Data and corresponding statistical analyses are from [30] and are reported here to show how changes in the endocannabinoidome induced by dietary ω-3 fatty acids is associated with metabolic changes.

### Effect of HF and KO on hepatic levels of AEA, 2-AG, OEA and PEA and biosynthetic precursors

**Figure 2:**
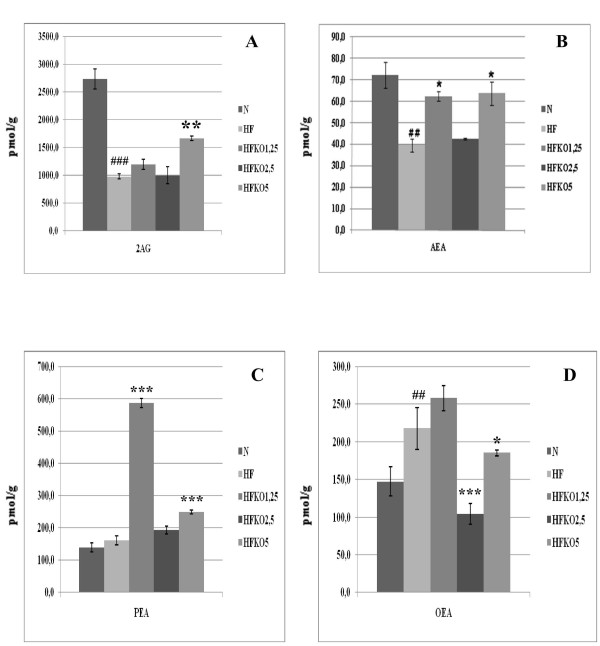
**Levels of 2-arachidonoylglycerol (2-AG, A), anandamide (AEA, B), *N*-palmitoylethanolamine (PEA, C) and *N*-oleoylethanolamine (OEA, D) in the liver of high-fat fed mice with dietary krill oil (HFKO) supplementation at different doses (1.25, 2.5 and 5% wt)**. Data are means ± SEM of separate determinations in N = 4-5 mice. ^## ^P < 0.01 and ^### ^P < 0.001 N vs HF; * P < 0.05 **P < 0.01 *** P < 0.001 high-fat diet (HF) vs HFKO1.25-2.5-5. N = normal diet.

Levels of AEA and 2-AG decreased significantly (P < 0.05 and P < 0.001) after HF diet, whereas OEA levels increased significantly (P < 0.01) and no significant difference was observed in PEA levels (Figure 2). KO supplementation increased AEA levels, but not in a dose-dependent manner and only at 1.25 and 5% dosages (P < 0.05). Instead, dietary KO increased 2-AG levels in a significant manner only at the highest dose of KO (HF *vs *HFKO5 P < 0.01). PEA levels increased significantly after KO supplementation at the lowest dose (HF *vs *HFKO1.25 P < 0.001) and decreased in a significant manner with the 2.5% and 5% doses (HF *vs *HFKO5 P < 0.001). Instead, OEA levels increased at the lowest dose of KO, were reduced significantly after HFKO2.5 (P < 0.001) and increased again at the highest dose of KO (P < 0.05).

The 2-AG biosynthetic precursors measured are the DAG 18:1-20:4, 16:0-20:4 and 18:0-20:4 (Table 4). They have the same basal levels in the liver and only the 18:1-20:4 DAG levels increased after HF diet, whereas in the other two species no differences were observed between N *vs *HF groups. Both 18:1-20:4 and 16:0-20:4 levels decreased after KO supplementation, whereas 18:0-20:4 levels increased in a significantly manner only at the highest dose of KO (P < 0.05).

**Table 4:** Diacylglycerol (DAG) levels in the liver, gastrocnemius mucle, heart, epididymal and inguinal adipose tissue and kidneys of high-fat fed mice with dietary krill oil (HFKO) supplementation

Tissue and DAGs	N (pmol/mg)	HF (pmol/mg)	HFKO1.25 (pmol/mg)	HFKO2.5 (pmol/mg)	HFKO5 (pmol/mg)
Liver					
18:1-20:4	10.8 ± 2.3	12.1 ± 2.8	7.7 ± 0.5	7.1 ± 1.9	7.3 ± 1.2

16:0-20:4	8.9 ± 2.4	8.1 ± 1.0	4.3 ± 1.0	4.2 ± 1.2	5.9 ± 1.7

18:0-20:4	7.6 ± 1.7	7.7 ± 1.3	11.2 ± 2.3	10.5 ± 2.4	17.4 ± 0.9*

Gastrocnemius					
18:1-20:4	25.0 ± 3.8	17.1 ± 3.3	11.5 ± 2.5	12.1 ± 3.3	17.7 ± 1.8

16:0-20:4	4.9 ± 1.1	8.7 ± 2.3^**#**^	3.4 ± 0.7*	4.2 ± 0.5*	3.7 ± 0.5*

18:0-20:4	43.6 ± 3.8	40.0 ± 7.0	27.1 ± 6.0	24.9 ± 2.1	20.4 ± 1.3*

Heart					
18:1-20:4	3.0 ± 0.2	7.2 ± 2.1	7.1 ± 0.7	5.1 ± 0.4	7.1 ± 1.5

16:0-20:4	0.4 ± 0.1	1.2 ± 0.4	0.5 ± 0.2	0.7 ± 0.2	0.4 ± 0.1

18:0-20:4	11.3 ± 0.6	21.1 ± 4.2^**#**^	11.8 ± 1.6^*^	13.2 ± 1.9^*^	10.1 ± 2.5^*^

Epididymal adipose tissue					
18:1-20:4	12.3 ± 1.9	37.0 ± 2.2^**###**^	4.3 ± 0.4***	4.7 ± 0.7***	2.9 ± 1.1***

18:0-20:4	13.9 ± 4.4	16.9 ± 2.2	37 ± 0.5***	6.8 ± 2.7*	3.0 ± 1.5**

Inguinal adipose tissue					
18:1-20:4	23.3 ± 3.26	16.3 ± 1.2^**#**^	9.7 ± 2.9	7.5 ± 1.4*	5.9 ± 0.5**

16:0-20:4	-	6.8 ± 0.8^**###**^	2.4 ± 0.4***	2.4 ± 0.4***	3.2 ± 0.2***

18:0-20:4	49.8 ± 12.6	31.3 ± 6.6	20.6 ± 2.2	12.5 ± 2.5	12.4 ± 1.6

Kidney					
18:1-20:4	2.0 ± 0.4	6.6 ± 1.4^**##**^	7.0 ± 0.7	3.2 ± 0.5*	5.1 ± 0.7

16:0-20:4	1.9 ± 0.1	1.3 ± 0.2	0.8 ± 0.2	1.1 ± 0.2	1.3 ± 0.2

18:0-20:4	10.4 ± 1.5	23.8 ± 5.3^**#**^	24.1 ± 1.4	21.3 ± 5.1	24.5 ± 1.3

Data are means ± SEM of separate determinations in N = 4-5 mice. ^# ^P < 0.05, ^## ^P < 0.01 and ^### ^P < 0.001 HF *vs *N; * P < 0.05, **P < 0.01 and *** P < 0.001 HFKO1.25-2.5-5 vs HF.

The AEA precursor, i.e. the NAPE with N-20:4 (NArPE, *N*-arachidonoylphosphatidyl ethanolamine), did not undergo any change (Table 5). No differences were observed in HF groups *vs *N for PEA precursor (NPPE, *N*-palmitoylphosphatidyl ethanolamine), like PEA, but dietary KO increased NPPE levels in a significantly way only at the highest dose of KO (P < 0.01). Instead, OEA biosynthetic precursor, *N*-oleoylphosphatidyl ethanolamine (NOPE), had exactly the same trend as OEA.

**Table 5:** Levels of *N*-arachidonoylphosphatidylethanolamine (NArPE), *N*-palmitoylphosphatidyl-ethanolamine (NPPE) and *N*-oleoylphosphatidylethanolamine (NOPE) in the liver, gastrocnemius mucle, heart, epididymal and inguinal adipose tissue and kidneys of high-fat fed mice with dietary krill oil (HFKO) supplementation

Tissue and NAPEs	N (pmol/mg)	HF (pmol/mg)	HFKO1.25 (pmol/mg)	HFKO2.5 (pmol/mg)	HFKO5 (pmol/mg)
Liver					
NPPE	1.2 ± 0.1	1.2 ± 0.1	1.7 ± 0.2	1.3 ± 0.1	2.0 ± 0.08**

NOPE	0.3 ± 0.02	0.4 ± 0.06	0.5 ± 0.06	0.3 ± 0.02	0.5 ± 0.04*

NArPE	0.2 ± 0.02	0.2 ± 0.06	0.2 ± 0.02	0.2 ± 0.03	0.2 ± 0.03

Gastrocnemius					
NPPE	1.1 ± 0.2	1.1 ± 0.08	0.9 ± 0.05	0.9 ± 0.09	0.7 ± 0.2

NOPE	0.8 ± 0.1	0.6 ± 0.04	0.5 ± 0.06	05 ± 0.06	0.4 ± 0.07

NArPE	0.3 ± 0.06	0.3 ± 0.05	0.1 ± 0.02*	0.06 ± 0.02**	0.04 ± 0.01**

Heart					
NPPE	1.7 ± 0.05	1.7 ± 0.2	1.8 ± 0.2	1.6 ± 0.1	1.5 ± 0.1

NOPE	0.2 ± 0.02	0.3 ± 0.01	0.3 ± 0.04	0.3 ± 0.03	0.2 ± 0.03

NArPE	0.2 ± 0.03	0.3 ± 0.05	0.2 ± 0.01	0.2 ± 0.03	0.1 ± 0.02**

Epididymal adipose tissue					
NPPE	3.6 ± 0.5	3.7 ± 1.2	1.6 ± 0.2	2.5 ± 0.4	1.9 ± 0.5

NOPE	2.9 ± 0.3	1.2 ± 0.1^**###**^	0.9 ± 0.2	1.1 ± 0.2	1.1 ± 0.3

NArPE	0.1 ± 0.02	0.3 ± 0.09^**#**^	0.07 ± 0.01*	0.1 ± 0.07*	0.04 ± 0.01*

Inguinal adipose tissue					
NPPE	2.8 ± 0.5	3.3 ± 0.8	2.1 ± 0.6	2.0 ± 0.4	1.5 ± 0.6

NOPE	3.1 ± 0.6	4.2 ± 1.1	2.3 ± 0.7	2.3 ± 0.4	1.6 ± 0.7

NArPE	0.3 ± 0.09	0.6 ± 0.2	0.4 ± 0.1	0.4 ± 0.03	0.3 ± 0.07

Kidney					
NPPE	1.2 ± 0.2	1.4 ± 0.2	1.4 ± 0.1	1.2 ± 0.12	1.1 ± 0.1

NOPE	1.1 ± 0.2	0.9 ± 0.2	0.8 ± 0.06	0.6 ± 0.05	0.5 ± 0.06

NArPE	1.1 ± 0.2	1.7 ± 0.2	1.0 ± 0.06	0.6 ± 0.7	0.5 ± 0.06

Data are means ± SEM of separate determinations in N = 4-5 mice. ^# ^P < 0.05, ^## ^P < 0.01 and ^### ^P < 0.001 N vs HF; * P < 0.05, **P < 0.01 and *** P < 0.001 HF vs HFKO1.25-2.5-5.

### Effect of HF and KO on the levels of AEA, 2-AG, OEA, PEA and biosynthetic precursors in gastrocnemius muscle

**Figure 3:**
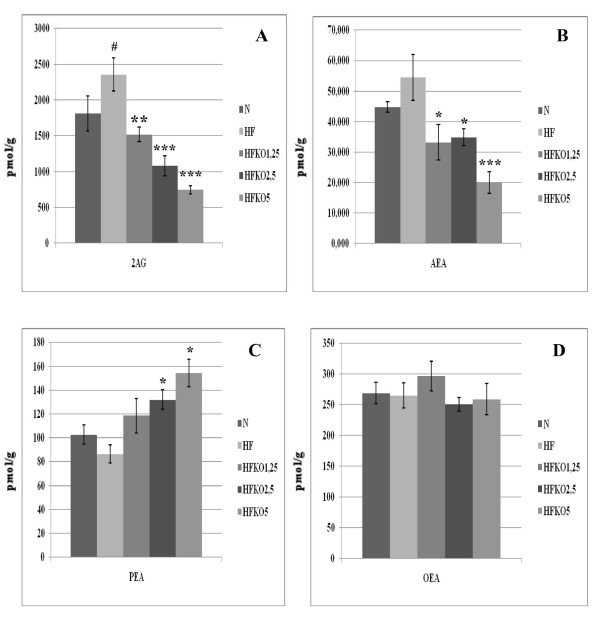
**Levels of 2-arachidonoylglycerol (2-AG, A), anandamide (AEA, B), *N*-palmitoylethanolamine (PEA, C) and *N*-oleoylethanolamine (OEA, D) in the gastrocnemius muscle of high-fat fed mice with dietary krill oil (HFKO) supplementation at different doses (1.25, 2.5 and 5% wt)**. Data are means ± SEM of separate determinations in N = 4-5 mice. ^# ^P < 0.05 N *vs *HF; * P < 0.05 **P < 0.01 *** P < 0.001 high-fat diet (HF) vs HFKO1.25-2.5-5. N = normal diet.

Levels of AEA and 2-AG increased in gastrocnemius muscle after the HF diet, but only the 2-AG level was significantly altered (P < 0.05, Figure 3). KO supplementation reduced AEA levels in a significant manner at all three doses (P < 0.05 HF vs HFKO 1.25-2.5, P < 0.001 HF *vs *HFKO5) and also reduced 2-AG levels in a dose-dependent manner (P < 0.01 HF vs HFKO 1.25, P < 0.001 HF *vs *HFKO2.5-5). Levels of PEA decreased non-significantly after the HF diet and increased significantly after KO supplementation (P < 0.05 HF *vs *HFKO2.5-5, Figure 2). No significant differences were observed in OEA levels (Figure 3).

Levels of 18:1-20:4 and 18:0-20:4 DAGs decreased after HF diet and tended to decrease after KO supplementation. Instead, 16:0-20:4 DAG levels showed the same trend as 2-AG levels, and increased after HF diet and decreased significantly at all three doses of dietary KO (P < 0.05, Table 4).

KO supplementation decreased NArPE levels but in a way that was not statistically significant (Table 5). Instead, no differences were observed in NPPE and NOPE levels with or without dietary KO supplementation (Table 5).

### Effect of HF and KO on the levels of AEA, 2-AG, OEA, PEA and biosynthetic precursors in heart

**Figure 4:**
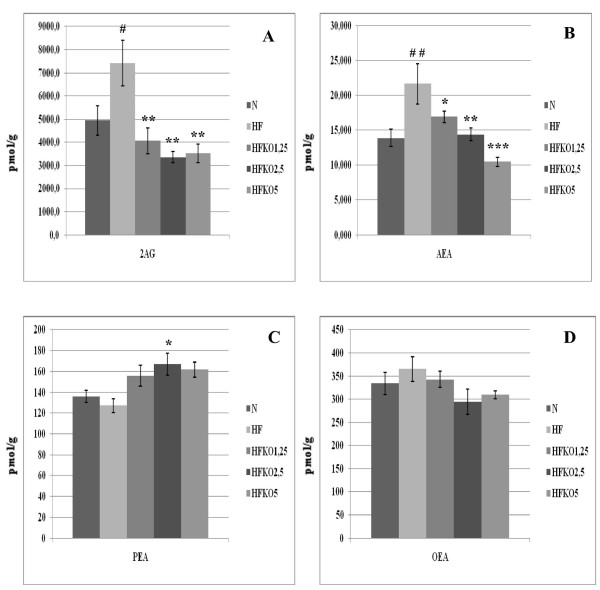
**Levels of 2-arachidonoylglycerol (2-AG, A), anandamide (AEA, B), *N*-palmitoylethanolamine (PEA, C) and *N*-oleoylethanolamine (OEA, D) in the heart of high-fat fed mice with dietary krill oil (HFKO) supplementation at different doses (1.25, 2.5 and 5% wt)**. Data are means ± SEM of separate determinations in N = 4-5 mice. # P < 0.05 and ## P < 0.01 N vs HF; * P < 0.05 **P < 0.01 *** P < 0.001 high-fat diet (HF) vs HFKO1.25-2.5-5. N = normal diet.

AEA and 2-AG levels increased after HF diet (P < 0.01 and 0.05) and decreased after KO supplementation, but only for AEA in a dose-dependent manner (Figure 4). Levels of PEA tended to decrease after HF diet and to increase after dietary KO, but in a statistically significant manner only at the 2.5% dose (P < 0.05, Figure 4). No differences were observed, instead, in OEA levels (Figure 4).

All three DAGs measured increased after HF diet, and tended to decrease in HFKO groups in a significantly way only for 18:0-20:4 DAG (Table 4).

NArPE levels showed the same trend as AEA levels (P < 0.05 N *vs *HF and HF *vs *HFKO1.25-2.5-5, Table 5). 

### Effect of HF and KO on the levels of AEA, 2-AG, OEA, PEA and biosynthetic precursors in epididymal and inguinal ATs

**Figure 5:**
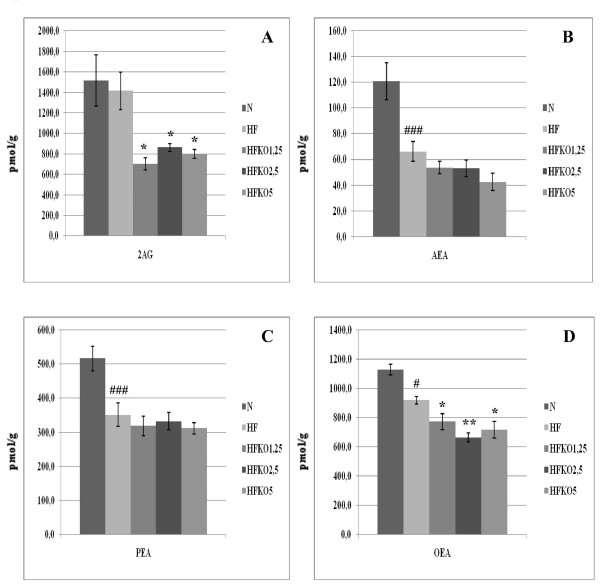
**Levels of 2-arachidonoylglycerol (2-AG, A), anandamide (AEA, B), *N*-palmitoylethanolamine (PEA, C) and *N*-oleoylethanolamine (OEA, D) in the epididymal adipose tissue of high-fat fed mice with dietary krill oil (HFKO) supplementation at different doses (1.25, 2.5 and 5% wt)**. Data are means ± SEM of separate determinations in N = 4-5 mice. ^# ^P < 0.05 and ^### ^P < 0.001 N vs HF; * P < 0.05 **P < 0.01 *** P < 0.001 high-fat diet (HF) vs HFKO1.25-2.5-5. N = normal diet.

**Figure 6:**
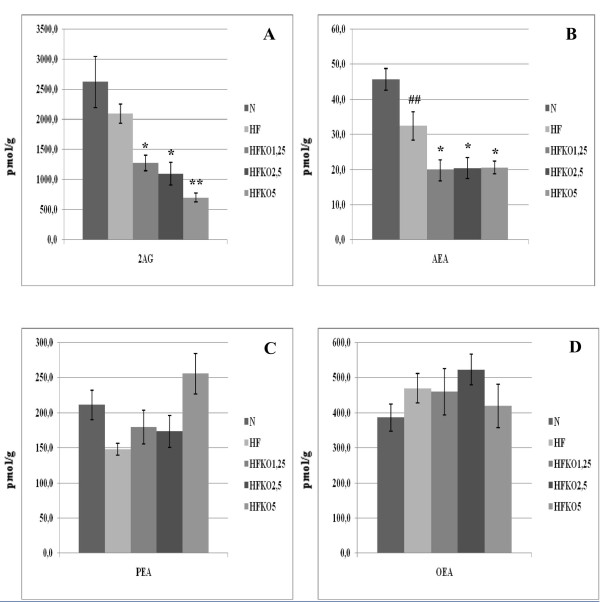
**Levels of 2-arachidonoylglycerol (2-AG, A), anandamide (AEA, B), *N*-palmitoylethanolamine (PEA, C) and *N*-oleoylethanolamine (OEA, D) in the inguinal adipose tissue of high-fat fed mice with dietary krill oil (HFKO) supplementation at different doses (1.25, 2.5 and 5% wt)**. Data are means ± SEM of separate determinations in N = 4-5 mice. ^## ^P < 0.01 N vs HF; * P < 0.05 and **P < 0.01 high-fat diet (HF) vs HFKO1.25-2.5-5. N = normal diet.

2-AG levels decreased after the HF diet in both AT depots and continued to decrease in HFKO groups, in a dose-dependent manner in the inguinal AT (P < 0.05 HF vs HFKO1.25-2.5, (Figure 5 and Figure 6) and in the same way at all three doses of HFKO in the epididymal AT (P < 0.05). Levels of AEA decreased after the HF diet in a statistically significant manner (P < 0.001 for epididymal and P < 0.01 for inguinal, Figure 5 and Figure 6) and decreased after KO supplementation in both ATs, but in a significant way only for inguinal fat (P < 0.05). No significant differences were observed in PEA and OEA levels in inguinal AT (Figure 6). In the epididymal fat, PEA levels decreased after HF diet (P < 0.001, Figure 5) and no further changes were observed in HFKO groups. OEA levels decreased in a significant way after HF diet (P < 0.05) and after KO supplementation (P < 0.05 HF *vs *HFKO1.25-5 and P < 0.01 HF *vs *HFKO2.5).

In the epididymal AT, of those DAG species we decided to measure, only the 18:1-20:4 and 18:0-20:4 species were quantifiable and both increased after HF diet and decreased in a dose-dependent and also statistically significant manner, for the 18:1-20:4 DAG (P < 0.001, Table 4), and in a non-dose-dependent manner for the other species. Instead, in the inguinal AT 18:1-20:4 DAG decreased after HF diet and decreased in a very significant manner in the HFKO groups (Table 4). The 16:0-20:4 species was not detectable in the N group and was observed only after KO supplementation (P < 0.001 HF *vs *all HFKO groups). Finally, no significant changes were observed for 18:0-20:4 DAG.

In the inguinal AT, NArPE levels did not change, whereas, in the epididymal fat, they increased after the HF diet and decreased in HFKO groups (Table 5). Both NPPE and NOPE in the inguinal fat did not undergo any changes (Table 5). In the epididimal AT, no differences were observed in NPPE levels, whereas NOPE levels decreased significantly after the HF diet (P < 0.001, Table 5).

### Effect of HF and KO on the levels of AEA, 2-AG, OEA, PEA and biosynthetic precursors in kidneys

**Figure 7:**
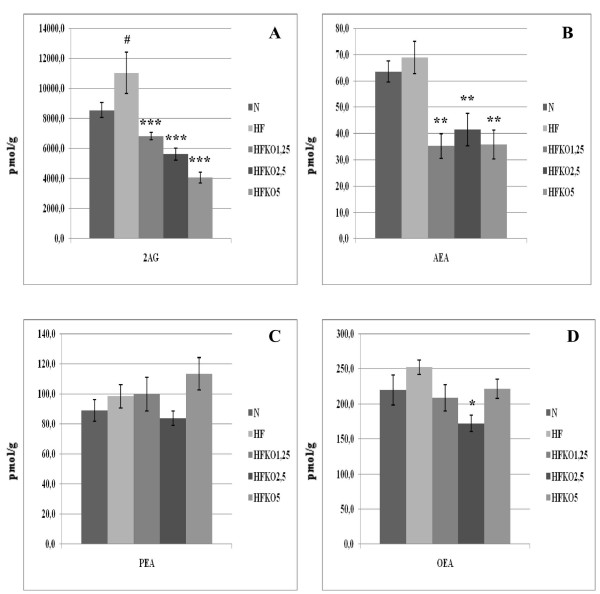
**Levels of 2-arachidonoylglycerol (2-AG, A), anandamide (AEA, B), *N*-palmitoylethanolamine (PEA, C) and *N*-oleoylethanolamine (OEA, D) in the kidneys of high-fat fed mice with dietary krill oil (HFKO) supplementation at different doses (1.25, 2.5 and 5% wt)**. Data are means ± SEM of separate determinations in N = 4-5 mice. ^# ^P < 0.05 N vs HF; * P < 0.05, **P < 0.01 and *** P < 0.001 high-fat diet (HF) vs HFKO1.25-2.5-5. N = normal diet.

AEA levels did not undergo any changes after HF diet but decreased in all HFKO groups in a very significant way (P < 0.01, Figure 7). Levels of 2-AG increased after HF diet and decreased in a dose-dependent manner after KO supplementation (Figure 7). No differences were observed in PEA levels, and OEA levels decreased in significant manner only with the middle dosage of KO (Figure 7).

Levels of both 18:1-20:4 and 18:0-20:4 DAG increased significantly after the HF diet and, whereas the first species increased at the lowest dose of KO and decreased at HFKO2.5 and 5, the other did not undergo any changes (Table 4). No differences were observed in 16:0-20:4 DAG levels. No significant changes were observed in NArPE, NPPE and NOPE levels (Table 5).

## Discussion

The dietary unbalance among macronutrients leads to metabolic derangement of glucose and lipid disposal characterised by a marked dyslipidemia, increased insulin resistance and fatty liver, which are some of the characteristic features of the metabolic syndrome. Whether not only the amount but also the composition of dietary fat may influence the development of the metabolic syndrome, mediated by the EC system, has been recently investigated [29]. In the present study, 8 week administration of a HF diet low in the n-6 PUFA precursor LA, led to a significant elevation of body weight and liver weight, as well as of hepatic TG, cholesterol and TNFα levels, and serum TG, cholesterol and PL levels. On the other hand, we did not observe induction of high fasting glycemia and hypoadiponectinemia, contrary to the findings previously obtained using a different type of HF diet, which had significantly higher LA content [28]. At the same time, the HF diet used here reduced, instead of increasing, EC levels in the liver, in contrast to what previously described using the other HF diet [28], which led to significantly enhanced hepatic AEA levels. The only explanation that we have for this discrepancy lies in the different n-6 PUFA precursor content of the HF diets used by us and Osei-Hyiaman and colleagues, who observed the strongest effect on AEA levels after 3 weeks of the dietary regimen. While these authors used partially hydrogenated oils (PHO), we used butter. These two fats have a dramatic different LA content (about 4% with PHO *vs *about 0.4% with butter). It has been pointed out before that the effect of HF diet on peripheral EC levels greatly depend on the fatty acid composition of the diet [33]. Chronic administration of a diet such as the one used in the current study, with a lower amount of LA than in a normal diet, may have caused a net decrease in hepatic AA, and hence in the levels of the ultimate PL biosynthetic precursors of ECs. However, as assessed by the "endocannabinoidomics" methodology used here, we could not see any decrease in the levels of AEA and 2-AG direct biosynthetic precursors following the HF diet, thus suggesting that strongly different fatty acid compositions of prolonged dietary regimens might affect EC levels also at other levels (see below). The higher amount of cholesterol in the HF diet used here is, instead, unlikely to have caused any effect on EC levels, since we have previously shown that an even more hypercholesterolemic diet affects tissue EC concentrations in mice only when it also provokes accumulation of atherosclerotic plaques [34].

Interestingly, a further change of dietary fat composition imposed with KO supplementation during the HF diet resulted in the elevation of both AEA (at both the lowest and highest dose) and 2-AG (only at the highest dose) hepatic levels, thus clearly dissociating the hepatic disorders accompanying HF diet-induced obesity from EC overactivity, at least with the type of HF diet used here. Previously, we had shown that in the liver of obese Zucker rats, in which elevated hepatic levels of both AEA and 2-AG occur [35], amelioration of hepatic steatosis following dietary KO was accompanied by a strong reduction of AEA levels, but also by an increase of the tissue concentration of 2-AG, which, in both species, is significantly more abundant than AEA. These data, taken together, might suggest that EC overactivity in the liver is not necessarily a determining factor for obesity-associated liver steatosis and underlying inflammation in rodents. On the other hand, contrary to the HF diet used by Osei-Hyiaman et al. [28], the dietary regimen employed here caused a reduction, rather than an increase, of two de novo lipogenesis-associated genes, FAS and ACC1, and this effect was potentiated by KO. These data can now be partly accounted for by the present finding of decreased or increased EC levels after the HF diet or the HF diet plus KO, respectively, and support the hypothesis that hepatic de novo lipogenesis is instead strongly sustained by ECs via CB_1 _receptor activation [28] but also counteracted by KO independently from the EC system. Clearly, obesity- and HF diet-associated-hepatosteatosis in mice is only part due to de novo lipogenesis in hepatocytes, and might be caused principally by other factors (such as fatty acid flux from the adipose tissue).

We found here that, contrary to EC levels, KO increases the levels of the PPARα activator, PEA, albeit in a non-dose-dependent manner. Given the beneficial effects of PPARα agonists on liver steatosis and inflammation [36], it is possible that the beneficial effects of KO in this case are due to its stimulatory action on this compound. However, KO reduced the levels of the other PPARα activator, OEA, thus possibly resulting in an overall null effect on PPARα activity. Importantly, like with the HF diet, also the effects of KO on hepatic EC, PEA and OEA levels did not appear to be largely accounted for by effects on the direct biosynthetic precursors of these compounds. Instead, the HF diet-induced increase of OEA levels and decrease in 2-AG levels might be explained by our previous finding [28] that this dietary regimen: 1) on the one hand, increases the expression of both CD36 and stearoyl-CoA desaturase, two proteins involved in the uptake and biosynthesis, respectively, of oleic acid, the ultimate precursor of OEA; and 2) on the other hand, increases also the expression of the monoacylglycerol lipase, the major enzyme involved in 2-AG degradation. These effects were significantly counteracted by KO [28], which, as mentioned above, also reduced and enhanced hepatic OEA and 2-AG, respectively, thus supporting the potential role of these proteins and enzymes in determining the levels of these two lipid mediators in the liver. KO at the highest dose, however, also increased the levels of 18:0-20:4-DAG, which corresponded to increased 2-AG levels.

Another major difference between the results of the present study and that of others [37], possibly attributable to the difference in dietary fat composition, was the fact that here the 8-week HF diet did not cause elevation of 2-AG levels in the epididymal AT (a type of adipose depot similar to intra-abdominal adipose tissue in humans), although it did provoke a reduction of EC levels in the inguinal AT similar to that observed in other subcutaneous adipose depots of both obese rodents [35,38] and humans [39,40]. The HF diet used here caused instead a significant reduction of AEA levels in the epididymal AT. Interestingly, unlike other types of HF diet, the one used here also did not induce high fasting glycemia or reduce serum adiponectin levels [30], thus strengthening the link between excessive EC tone in the visceral AT, or lack thereof, and insulin resistance or sensitivity, respectively. In this case, the relatively low levels of LA present in our HF diet are unlikely to have caused an increase of AA in PL biosynthetic precursors of ECs, since both NArPE and *sn*-2-arachidonoyl-containing DAGs were increased, rather than decreased, by the HF diet. Despite the lack of stimulatory effect of the HF diet on epididymal AT EC levels, dietary KO strongly reduced 2-AG levels in the epididymal AT, and also down-regulated both AEA and 2-AG levels in the inguinal AT, whilst reducing fasting glycemia and elevating adiponectin levels. These data, taken together with the little effect of KO on PEA or (with the exception of the epididymal AT) OEA levels, suggest that EC tone in the white AT, rather than in the liver, might be an important determinant of both insulin resistance and ectopic (hepatic) fat formation. The lowering of AT EC levels by dietary KO might, therefore, represent an interesting alternative to CB_1 _receptor antagonists to counteract the development of pre-diabetes and hepatosteatosis. Importantly, unlike the effects exerted by the HF diet *per se*, the alterations (or lack thereof) caused by KO on EC, PEA and OEA levels in both AT depots analysed here could be mostly accounted for by similar effects on the direct biosynthetic precursors of these mediators.

Excessive EC levels in the skeletal muscle was suggested as another potentially important cause of high fasting glycemia [33]. In fact, activation of CB_1 _receptors in myotubes modifies insulin signalling, reduces sensitivity to insulin and impairs glucose uptake [41]. Furthermore, elevation of EC levels triggers an inhibition of AMPK1α and, subsequently, of glucose uptake and oxidation from the skeletal muscle [42]. Here we found, in the gastrocnemius skeletal muscle of HF diet-fed mice, a significant elevation of 2-AG levels. Likewise, in a previous study, Matias and collaborators [33] found an increase of 2-AG levels after a 3-week regimen with another type of HF diet and, although this effect was not significant at 8 weeks of regimen, it was observed still after 14 weeks. More importantly, we observed here that dietary KO supplementation decreased 2-AG levels in a dose-dependent manner and also reduced the levels of one of the DAGs analysed, suggesting a beneficial role of KO at enhancing energy expenditure and reducing fasting glucose levels. Furthermore, we observed that KO supplementation was accompanied also by elevation of PEA levels. Given the role of PEA as anti-inflammatory agent [43], and the previous observation that omega-3 PUFAs exert a protective effect against muscle damage induced by the pro-inflammatory cytokine TNF-α [44], we speculate that increased PEA levels might protect skeletal muscle from the damaging effect of TNF-α, and contribute, together with KO-induced (an possibly AT EC decrease-mediated) elevation of adiponectin levels, to the anti-inflammatory effects of KO. Our "endocannabinoidomic" profiling data also indicate that the changes caused by HF diet or KO on EC levels in the skeletal muscle are mostly accounted for by similar effects on the direct biosynthetic precursors of these mediators.

Another novel observation of this study was the finding that dietary KO during a HF diet can counteract the effect of the latter on heart EC levels. HF-induced elevation of cardiac EC levels was observed also previously using a different HF diet [33], and we have shown here that KO dose-dependently reduces AEA levels, and is effective at re-establishing normal 2-AG levels already at the lowest dose tested, whilst producing little, if any, effect on PEA and OEA levels. ECs directly affect cardiac function by acting at CB_1 _and CB_2 _receptors and, in the case of AEA and OEA, potentially also via TRPV1 receptors [45]. Although the subsequent overactivity of CB_1 _receptors might enhance lipogenesis in the heart, thus contributing to formation of pericardial fat, which is a risk factor for cardiovascular events [33], the endocannabinoid system may also be activated as a compensatory mechanism in various forms of hypertension to limit pathologically increased blood pressure and myocardial contractility [46]. In obese Zucker rats, KO was previously shown to reduce cardiac AEA levels, whilst elevating 2-AG levels, and concomitantly reducing TG accumulation in the heart. Although we did not measure here fat accumulation in the heart of HF-treated mice, nor the amount of pericardial AT, we can speculate that KO-induced reduction of cardiac EC levels might contribute to reducing ectopic fat formation in this organ, and hence cardiovascular risk. Our "endocannabinoidomics" results also suggest that the decrease of EC levels carried about by KO can be due to lesser availability of EC biosynthetic precursors.

In 2007, Janiak and collaborators showed that chronic blockade of CB_1 _receptors could reduce renal failure caused by obesity and type 2 diabetes in Zucker rats in a way partly independent from inhibition of food-intake [47]. Furthermore, in an another study Matias et al. demonstrated that 8-week HF fed mice caused a strong elevation of AEA levels, possibly contributing to decreased glomerular filtration rate and increased renal blood flow typical of obesity [32]. In the present study, both AEA and 2-AG levels increased after the HF diet, although only 2-AG in a statistically significant manner. Importantly, the kidney levels of both compounds were strongly decreased after KO supplementation, which instead did not so strongly affect OEA and PEA levels. Since renal EC overactivity could contribute to kidney malfunctioning and cardiovascular disease, in part due to renal AT formation [48], KO could be a promising alternative to pharmacologic treatment for the renal complications of obesity and the metabolic syndrome. Importantly, whilst the effects of KO on kidney 2-AG levels were reflected by its effects on the DAG precursors of this compound, KO supplementation did not significantly affect the levels of the direct AEA biosynthetic precursor, NArPE, although a trend towards its decrease was observed.

In conclusion, our data have shown that also in mice with HF-induced obesity and dysmetabolism, n-6 PUFAs dietary content influences EC precursors and biosynthesis, and that the addition of KO to the diet can ameliorate several metabolic disturbances and reduce EC levels in most of those peripheral organs the malfunctioning of which is responsible for such disturbances. Furthermore, by using a novel lipid profiling methodology, we have confirmed the hypothesis according to which KO-induced reduction of the levels of AEA and/or 2-AG is at least in part due to reduction of the tissue levels of the corresponding direct precursors of these mediators. Finally, again by carrying out the simultaneous quantification of *N*-acylethanolamine activators of PPARα, such as PEA and OEA, we have shown that KO affects the levels of these mediators, although only in some of the analysed tissues. Since these two mediators are dysregulated in several tissues of obese Zucker rats [49], these data might suggest that KO can potentially produce beneficial metabolic effects against dysmetabolism and inflammation in obesity also by re-equilibrating the activity of PPARα.

## Conflict of interest

Aker Biomarine ASA, Norway, funded part of this study. KB is an employee of Aker Biomarine ASA, Norway.

## Authors' contributions

FP, GC and TB carried out the measurement of endocannabinoids and lipid congeners and their biosynthetic precursor levels and performed the statistical analysis. FB and VD drafted the manuscript. KB, ST and JC provided animals and krill oil. GC, EM, LC and MG participated in the study of the lipid and nutrient composition of the diet. VD and SB conceived of the study, and participated in its design and coordination. All authors read and approved the final manuscript.
